# Beyond latitude: thermal tolerance and vulnerability of a broadly distributed salmonid across a habitat temperature gradient

**DOI:** 10.1093/conphys/coaf030

**Published:** 2025-05-01

**Authors:** Terra L Dressler, Kara Anlauf-Dunn, Andrea Chandler, Erika J Eliason

**Affiliations:** Department of Ecology, Evolution, and Marine Biology, University of California, Santa Barbara, CA 93106, USA; Stillwater Sciences, 996 S. Seaward Ave, Suite 102, Ventura, CA 93001, USA; Conservation and Recovery, Oregon Department of Fish and Wildlife, 28655 Highway 34, Corvallis, OR 97333, USA; Department of Ecology, Evolution, and Marine Biology, University of California, Santa Barbara, CA 93106, USA; Conservation and Recovery, Oregon Department of Fish and Wildlife, 28655 Highway 34, Corvallis, OR 97333, USA; Department of Ecology, Evolution, and Marine Biology, University of California, Santa Barbara, CA 93106, USA

**Keywords:** Aerobic scope, climate change, field respirometry*, Oncorhynchus mykiss*, wild fish

## Abstract

Salmonid fishes are a focal point of conservation physiology due to their high value to humans and ecosystems, and their susceptibility to decline from climate change. A significant challenge in conserving these fishes is that populations of the same species can be locally adapted to vastly different habitats within their wild ranges and can therefore have unique tolerance or vulnerability to environmental stressors within those habitats. Within the state of Oregon, USA, summer steelhead (*Oncorhynchus mykiss*) inhabit both cool, coastal waters most typically associated with Pacific salmonids and arid, inland environments where temperatures are more extreme. Here, we utilized streamside physiological experiments paired with habitat temperature monitoring to assess the thermal tolerance and vulnerability of four populations of summer steelhead from distinct thermal habitats. All populations had unique responses of critical thermal maximum, aerobic scope and exercise recovery to temperature. Despite populations from warm habitats exhibiting higher thermal tolerance than populations from cooler habitats, summer steelhead from warm habitats appear to be more vulnerable to the physiological consequences of warming based on the extreme temperatures they already experience during the summer. These results demonstrate an example of thermal physiology varying between populations within the same portion of their latitudinal range and highlight the need for habitat-specific conservation strategies for this species.

## Introduction

Aquatic environments face intensifying pressures from the effects of global climate change including increases in average water temperatures, daily and seasonal thermal variability and the magnitude and frequency of episodic heat waves (Ficke *et al.,* 2007; Rijnsdorp *et al.,* 2009; Kaushal *et al.,* 2010; Reid *et al.,* 2019). Warming directly challenges the survival and fitness of fishes due to physiological disruptions at the biochemical, tissue/organ and whole organism levels (Schulte *et al.,* 2011; Whitney *et al.,* 2016; Little *et al.,* 2020b). Fishes can respond to temperature challenges via thermal acclimation processes (i.e. changing morphology and physiology to improve performance under new conditions; Seebacher *et al.,* 2015), and in the context of a highly variable environment, responding quickly (i.e. within hours or days) may be critical (Sidell *et al.,* 1973; Hazel and Prosser, 1974; Sandblom *et al.,* 2014; Johansen *et al.,* 2021). Physiological thermal tolerance limits and habitat temperature patterns can be used to elucidate the vulnerability of fish species to climate warming. When a species inhabits a broad geographic range, however, genetically distinct populations can experience vastly different thermal conditions and exhibit interpopulation variability in thermal tolerance (Fangue *et al.,* 2006; Barrett *et al.,* 2011; Eliason *et al.,* 2011; Narum and Campbell, 2015; Zillig *et al.,* 2021). This makes it challenging to understand which populations are most vulnerable to warming and to decide where more active management actions should be taken.

Numerous studies have detected interpopulation variation in thermal tolerance within species of fish, where populations occupying warmer habitats can withstand higher temperatures than populations inhabiting cooler habitats (McKenzie *et al.,* 2021). In some cases, this variation follows a latitudinal gradient, such as in Atlantic killifish (*Fundulus heteroclitus*) where a subspecies in the warmer, southern portion of the species’ range has a higher critical thermal maximum (CT_MAX_) and mitochondrial oxygen binding capacity than a subspecies inhabiting the cooler, northern part of the range (Fangue *et al.,* 2009; Chung *et al.,* 2017). However, intraspecific variation in thermal tolerance can exist on an even finer scale given large enough differences in habitat temperatures with limited gene flow between habitats. Adult sockeye salmon (*Oncorhynchus nerka*) populations have differing optimal temperature windows for aerobic and cardiac function that closely correspond with their natal stream temperatures within a single watershed (Fraser River) in British Columbia (Eliason *et al.,* 2011). Embryo and juvenile *O. nerka* in the same system have different optimal rearing temperatures, swimming performance temperatures and critical thermal limits based on the temperatures of their rearing habitats (Chen *et al.,* 2013; Whitney *et al.,* 2013, p. 20; Eliason *et al.,* 2017). European perch (*Perca fluviatilis*) inhabiting a chronically warm enclosure near a power plant exhibit thermal compensation of resting oxygen uptake and heart rates as well as increased mitochondrial capacities compared to a reference population inhabiting cooler temperatures (Sandblom *et al.,* 2016; Pichaud *et al.,* 2019). Redband trout (*Oncorhynchus mykiss gairdneri*) from a desert population have been found to have a higher upper thermal limit, broader optimum temperature window for aerobic scope, higher maximum heart rate and reduced heat shock protein expression following exposure to diel thermal stress compared to a montane population when reared in common garden conditions (Narum *et al.,* 2013; Chen *et al.,* 2018). Understanding patterns of intraspecific variation in thermal requirements is critical for effective management of fish populations in the face of climate change.

Intraspecific variation in thermal tolerance can be assessed using both critical and functional thermal limit tests, each of which has benefits and limitations. Critical thermal maximum (CT_MAX_), the temperature where fish lose equilibrium when temperature is increased rapidly, acts as a proxy for lethal thermal limits (Beitinger and Lutterschmidt, 2011). Habitat temperature maximums can be subtracted from CT_MAX_ to determine thermal safety margins (TSMs) for each population (Sunday *et al.,* 2014; Pinsky *et al.,* 2019). CT_MAX_ tests are relatively easy and quick to perform and have been conducted on countless fish species to date, facilitating comparisons across and within species. However, many essential and fitness-enhancing functions become limited at temperatures below CT_MAX_ (Rodnick *et al.,* 2004; Farrell, 2009; Eliason *et al.,* 2022), and these tests consequently do not allow for predicting the onset of thermal stress or for identifying optimal habitat temperatures.

Functional thermal tolerance can be assessed by determining the upper thermal threshold when key physiological performance metrics become impaired. Fish require energy for maintenance (e.g. circulation, respiration, nervous function, protein turnover), growth (tissue biosynthesis) and for performance functions such as feeding, digestion and predator evasion that are essential for long-term survival and fitness (Fry, 1971; Claireaux and Lefrançois, 2007; Farrell, 2009; Eliason *et al.,* 2022). Recovery from exhaustive exercise is an ecologically important factor for fishes, given that many fish rely on anaerobic exercise to catch prey, escape predators and compete with conspecifics (Birnie-Gauvin *et al.,* 2023), yet are vulnerable (e.g. to predation, disease) and may miss opportunities (e.g. feeding, mating) during the recovery period. Both the energetic cost and duration of recovery can increase with warming (Kraskura *et al.*, 2020). Absolute aerobic scope (AAS) is the energetic capacity to support activities beyond maintenance at a given temperature and is calculated by subtracting a fish’s oxygen uptake rate at rest (resting metabolic rate [RMR]) from its maximum capacity for oxygen uptake (maximum metabolic rate [MMR]; Farrell, 2009). Factorial aerobic scope (FAS = MMR/RMR) is the factor by which an individual can increase metabolism above maintenance levels to support the costs of physiological functions (e.g. digestion, locomotion; Careau *et al.,* 2014). FAS can indicate when a metabolic constraint begins to develop. AAS and FAS tend to decrease at high temperatures because RMR tends to increase exponentially with temperature while MMR typically cannot increase past a certain temperature (Fry, 1947; Farrell, 2016; Eliason *et al.,* 2022). *Oncorhynchus mykiss* require an FAS of at least 2 (the ability to double RMR) in order to digest a moderate-sized meal, and it is estimated that they require an FAS of at least 3 (the ability to triple RMR) to be able to perform other functions during digestion (Eliason *et al.,* 2008, 2022; Adams *et al.,* 2022). The difference between the temperature where FAS = 3 (T_FAS3_) and the maximum stream temperatures can be used to calculate the functional warming tolerance (FWT) for a given juvenile trout population (Anlauf-Dunn *et al.,* 2022; Eliason *et al.,* 2022). This represents the amount of warming a stream can undergo before the fish experience functional limitations, which is useful for informing management actions such as habitat restoration and angling restriction.

Herein, we determine the thermal tolerance and vulnerability of four populations of a broadly distributed fish species, *O. mykiss*, occupying different thermal environments within the state of Oregon, USA. *Oncorhynchus mykiss*, also known as steelhead (anadromous phenotype) or rainbow trout (freshwater resident phenotype), naturally occur along the west coast of North America from southern California to Alaska and inhabit a wide range of thermal conditions both between and within latitudes (Page and Burr, 2011). In Oregon, populations with the potential to express the anadromous phenotype (i.e. they have access to the ocean) are often referred to colloquially and by managers as ‘steelhead’ in order to distinguish them from populations comprised entirely of the freshwater resident phenotype. The populations studied herein all contain anadromous individuals, so we refer to them as ‘steelhead’ for the remainder of this manuscript. This study is focused on summer-run steelhead, or the stream-maturing phenotype of steelhead (as opposed to the later returning, ocean-maturing winter steelhead) that inhabit both cool, coastal watersheds and arid, inland watersheds within throughout the state of Oregon. Individuals of this ecotype migrate as immature adults from the ocean during the summer and remain in freshwater until spawning in winter and early spring. Summer-run steelhead are listed as ‘threatened’ under the Endangered Species Act in many watersheds throughout Oregon, and there are active management efforts to conserve and protect them (National Marine Fisheries Service, 2009; Oregon Department of Fish and Wildlife, 2011). We focused on juveniles that remain in tributaries for the entirety of this life stage and must be able to survive summer temperatures to reach adulthood. We conducted streamside thermal tolerance experiments on field-acclimatized individuals to assess the ability of these fish to rapidly respond to increasing temperatures. These field-based experiments allow us to obtain a more realistic picture of the physiological response to warming than would a more traditional approach where fish are acclimated to lab conditions over several weeks. The goals of this study were (1) to compare thermal tolerance between populations of juvenile summer-run steelhead inhabiting different thermal regimes within the same part of their latitudinal range and (2) to pair thermal tolerance and habitat temperature data to determine which populations are currently most vulnerable to decline or extirpation from rising temperatures. We hypothesized that populations of summer-run steelhead from warm habitats have higher critical and functional thermal tolerance compared to populations from cool habitats. We also hypothesized that populations from warm habitats are more vulnerable to warming (i.e. have lower TSMs and FWT) because current temperatures are closer to their thermal limits during the summer.

## Materials and Methods

We tested the functional and critical thermal tolerance of wild juvenile summer-run *O. mykiss* from four watersheds located throughout the state of Oregon during July and August of 2021 and 2022. All experiments were conducted streamside, and each population was tested in its natal water. Streamside experiments are advantageous because they allow for close mimicking of natural temperature conditions, minimization of transport stress and release of fish back into the wild after testing. In this study, wild-caught fish were acclimatized to local field conditions, and each population was expected to be genetically distinct given that they are from different watersheds (Arciniega *et al.,* 2016), though genetic analysis was not conducted to confirm. Accordingly, any differences between watersheds may be due to a combination of genetic differentiation (e.g. local adaptation) and plasticity (e.g. developmental plasticity, parental effects, acclimatization). All methods were approved by the University of California Santa Barbara Institutional Animal Care and Use Committee (Protocol #928.1).

### Life history

We studied *O. mykiss* in locations that are known to specifically support summer-run steelhead and not winter-run steelhead. Winter-run and summer-run steelhead are physically indistinguishable, but in most cases, there is natural run differentiation and genetic differences between the two ecotypes (Papa *et al.,* 2007; Arciniega *et al.,* 2016). Winter-run steelhead cannot access our selected study locations due to flow conditions at the times that they migrate. Given the vastly different geographies of our study locations, the life history and phenological timings are variable by basin. In general, adults migrate to their freshwater spawning grounds after 2–4 years at sea between May and October each year. Peak migration timing varies depending on the location with peaks occurring May through July in western Oregon basins (Siletz and North Umpqua), and July and August for eastern Oregon basins (e.g. Lower Deschutes and John Day). Adult steelhead hold in freshwater until spawning, which occurs between January and May. Peak spawning timing varies by basin with earlier peaks seen in the western basins. Juvenile summer steelhead rear in freshwater for 1–3 years. Smolt outmigration occurs between January and June and peaks during late spring. Experiments were conducted on juvenile fish that were likely 1–2 years old.

### Populations

**Figure 1:**
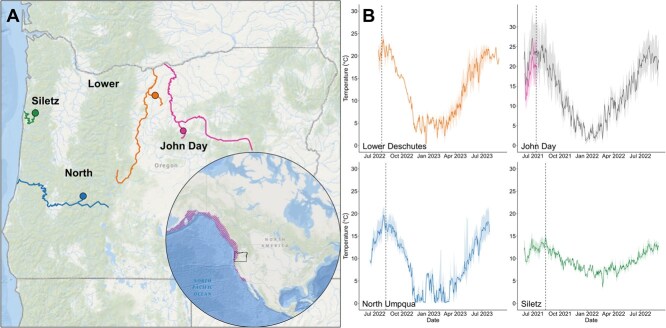
Map of the study area (**A**) including study streams (shown as coloured lines), exact study locations (represented by circular points) and the state of Oregon, USA, in relation to the latitudinal distribution for native steelhead (shown in the circular panel; AquaMaps, 2019). (**B**) Continuous temperature data collected from each study location in the weeks leading up to the experiments and for 1 year after the experiments. Vertical dotted lines indicate the first day of experiments. Solid lines represent mean daily temperatures, and shaded areas show the daily temperature range. For the John Day study location, the data shown in pink were measured by our logger, and the data shown in grey were measured by the United States Geological Survey (gauge #14046778) located ~20 km downstream of our study site.

We selected four populations as the focus of this study and tested fish residing in third-order or fourth-order tributaries within each system. Two populations (Lower Deschutes and John Day) were from interior streams characterized by high and variable summer temperatures (indicated throughout in warm colours; Fig. 1). The other two populations (North Umpqua, Siletz) were from more temperate, coastal river systems (indicated throughout in cool colours; Fig. 1).

#### Lower Deschutes

Buckhollow Creek is a fourth-order tributary of the Deschutes River in the eastern portion of the Lower Deschutes basin (Fig. 1). The land ownership and management of the Lower Deschutes is primarily private with federal (United States Bureau of Land Management) lands along the river and the stream corridors. The watershed is characterized by Columbia River basalt flows and has a semiarid climate. Buckhollow Creek has a narrow riparian corridor of mostly willow, cottonwood and alder and various grass species. Past land use practices have altered riparian structure and reduced floodplain connectivity. Summer temperatures are relatively warm in Buckhollow Creek, ranging from ~16°C to 25°C (Table 1 and Fig. 1).

**Table 1:** Environmental characteristics for each study location

	Lower Deschutes (Buckhollow Creek)	John Day (Bridge Creek)	North Umpqua (Steamboat Creek)	Siletz (Gravel Creek)	Source
Watershed size (km^2^)	512.8	697.32	588.51	24.89	n/a
Steelhead distribution area (km^2^)	115.59	62.02	82.67	9.82	n/a
Migrating distance (km)	428.29	561.32	260.06	107.71	n/a
Estimated mean annual discharge (m^3^ s^−1^)	71.8	35	77	11.7	Liang *et al.,* 1994
30-year annual precipitation range (mm)	312–341	303–839	1239–2050	2008–3825	Daly *et al.,* 1994
2021 annual estimate of precipitation (mm)	219–240	185–590	987–1720	2288–4306	Daly *et al.,* 1994
Elevation (m)	229	1930	580	211	n/a
Annual temperature range	~1–25°C	~1–27°C	~1–21°C	~2–16°C	Present study
Maximum diurnal temperature range	7°C	13°C	6°C	4°C	Present study

#### John Day

Bridge Creek is a fourth-order tributary of the John Day River in the Lower John Day basin in eastern Oregon (Fig. 1). The majority of Bridge Creek is under federal ownership (United States Bureau of Land Management), though the upper third of the watershed is private. While surrounded by volcanic lithologies, Bridge Creek is primarily overlain by sedimentary and plutonic lithologies, residing within the Calarno unit, which contains fossil bearing rock formations (Bestland *et al.,* 2002). The riparian corridor consists of mostly willow, juniper and sage. Past land use practices (e.g. grazing) have altered the vegetation structure. In the winter, much of the precipitation arrives as snow. Summer temperatures are warm in Bridge Creek, ranging from ~9°C to 27°C (Table 1 and Fig. 1).

#### North Umpqua

Steamboat Creek is a third-order tributary of the North Umpqua River in Western Oregon (Fig. 1). The Steamboat creek watershed is under federal (United States Forest Service) land ownership and management and is underlain by volcanic lithology. The riparian corridor consists of alder and maple with dense conifer forests adjacent along upland slopes. Precipitation arrives primarily in the form of snow. Summer temperatures are intermediate in Steamboat Creek, ranging from ~8°C to 21°C (Table 1 and Fig. 1).

#### Siletz

Gravel Creek is a third-order tributary of the Siletz River in Western Oregon (Fig. 1). The Gravel creek watershed has private industrial timber as the primary land ownership/management and is underlain by sandstone and basalt. The riparian corridor consists of primarily alder, big leaf maple and secondary growth conifer species. The climate is highly influenced by the climate patterns of the Pacific Ocean and the majority of the precipitation falls as rain in the winter months. Summer temperatures are relatively cool in Gravel Creek, ranging from ~8°C to 16°C (Table 1 and Fig. 1).

### Habitat temperature monitoring

We deployed Onset HOBO TidbiT MX temperature data loggers in each stream to collect continuous measurements of water temperature once per hour. Data loggers were installed at the bottom of each stream in the head or tail of pools where water was fast flowing and well mixed. Loggers were deployed before the start of the experiment and measured for the duration of the experiment and for 1 year afterwards with the goal of capturing the maximum temperatures, as well as the daily temperature variability, that each population experiences during the summer. Maximum stream temperatures recorded during this time were used in TSM and FWT calculations. The John Day logger was washed away during winter storms after the experiment, so maximum stream temperatures and daily variability were calculated from the data recorded prior to and during our experiments. John Day temperature data plotted in Fig. 1 in grey is from a gauge deployed by the United States Geological Survey (USGS) located ~20 km downstream of our study location (gauge #14046778). According to this USGS dataset, the temperature data from our John Day site appears to have been recorded during the warmest period within 1 year of the study (Fig. 1).

### Experimental setup and holding temperatures

**Figure 2:**
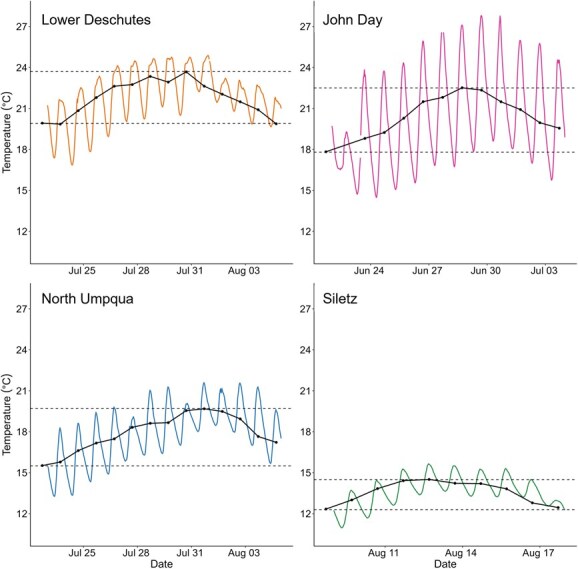
Examples of heat wave events in each study stream. Coloured lines show thermographs from start to end of each example heat wave, as measured by HOBO TidbiT MX loggers. Points indicate mean daily temperatures. Dashed lines indicate minimum and maximum mean daily temperature values over the course of each heat wave.

**Figure 3:**
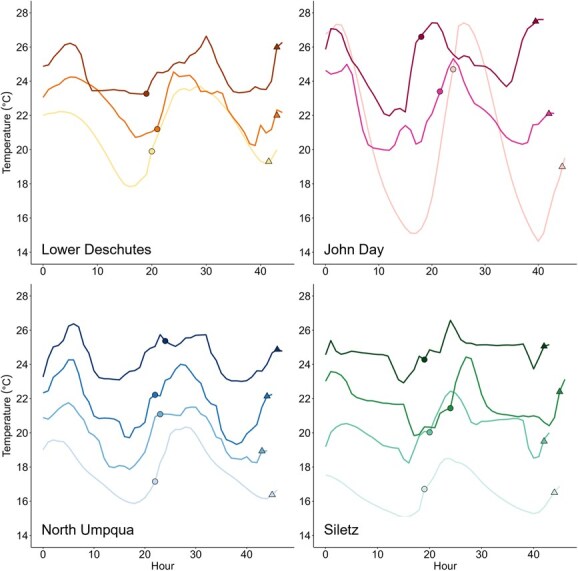
Holding and experimental temperature exposures for each population. Data are shaded to differentiate holding temperature treatments, with ambient treatments as the lightest colours and the warmest treatments as the darkest colours. All temperature traces start at the beginning of the acclimation treatment. For ambient treatments, traces start at the time when fish were placed into the acclimation tank. For all other treatments, traces start when fish have been placed in the acclimation tank and ramped up to treatment temperatures at a rate of ~2°C per hour. Circular points show the time point when fish were transferred into respirometers and RMR measurements began. Triangular points represent the time that MMR measurements occurred. Temperature traces end at the time point when exercise recovery measurements were complete. Critical thermal maximum (CT_MAX_) trials occurred shortly thereafter.

At each site, we constructed a temporary partially recirculating tank system pumping water from the stream through a series of tanks used for temperature exposure and respirometry. Juveniles from each *O. mykiss* population (Lower Deschutes: *n* = 33, mean ± SEM body mass = 15.60 ± 1.89 g; John Day: *n* = 39, mean ± SEM body mass = 38.7 ± 2.34 g; North Umpqua: *n* = 43, mean ± SEM body mass = 17.49 ± 1.06 g; Siletz: *n* = 40, mean ± SEM body mass = 24.01 ± 1.27 g) were captured via electrofishing and exposed to one of three or four fluctuating temperature treatments for 20 hours prior to physiological testing (Fig. 3). While some aspects of thermal acclimation can occur rapidly in fishes (Klicka, 1965; Barrionuevo and Fernandas, 1998; Macnutt *et al.,* 2004; Gilbert *et al.,* 2022), this 20-hour exposure is relatively acute and is likely not enough time for the fish to complete a full acclimation response (Stewart *et al.,* 2023). Due to the stochastic nature of temperature in these systems and the speed at which temperature can increase during heat waves (Fig. 2), this acute exposure was more ecologically relevant than allowing the fish several weeks to acclimate, as is typical in lab studies. Fish were not fed during holding to ensure that they would not be digesting during thermal tolerance experiments, as digestion introduces additional energetic costs (McCue, 2006; Eliason *et al.,* 2008). Holding and respirometry tanks were covered with mesh cloth and shade canopies to ensure that food items did not fall into the tanks.

Temperature fluctuates diurnally during the summer months for all four populations, between 4°C and 5°C for the Deschutes, Siletz and North Umpqua populations, and up to 14°C for the John Day population (Figs 1 and 2). We therefore used fluctuating treatments in our experiments to ensure that we were mimicking the fish’s natural environment as closely as possible (Fig. 3). All populations were tested at ambient temperatures, plus two to three warmer treatments mimicking the maximum temperatures experienced in their habitat and/or realistic potential future temperature scenarios under climate change (Fig. 3). For the Siletz population, temperature treatments included ambient temperatures (15–18°C), 18–22°C, 20–24°C and 23–26°C. For the North Umpqua population, treatments included ambient temperatures (16–20°C), 18–22°C, 20–24°C and 23–26°C. For the Lower Deschutes population, treatments included ambient temperatures (18–22°C), 20–24°C and 23–27°C. For the John Day population, treatments included ambient temperatures (14–27°C), 20–24°C and 23–27°C. Thus, common temperature treatments included 18–22°C (three out of four populations), 20–24°C (all populations) and 23–26/27°C (all populations).

### Functional thermal tolerance: aerobic scope and exercise recovery

We used intermittent flow respirometry to measure oxygen uptake rates (MO_2_) during rest and immediately after exercise (see Table S1 for full details). To measure MO_2_, individual fish were placed in a watertight plastic container (Lock & Lock, Seoul, South Korea) fitted with a FireStingO2 robust oxygen probe (PyroScience, Germany) to measure dissolved oxygen and a Universal 300 L h^−1^ aquarium pump (Eheim, Germany) to circulate water throughout the chamber. A MICRA Compact 90 GPH aquarium pump (SICCE, Italy) flushed oxygenated water through each chamber from the surrounding tank between MO_2_ measurements so that the fish never experienced dissolved oxygen levels below 80% air saturation. During each trial, one chamber was left empty to measure bacterial respiration, which was found to be negligible in all cases. Fish remained in good condition throughout respirometry trials with the exception of one mortality that occurred during the warmest trial for the Siletz population.

MO_2_ was measured for 20 hours to obtain RMR measurements for each temperature during the diurnal cycle. All trials used a 6-minute measurement period followed by a 4-minute flush cycle for RMR measurements with two exceptions: extra time was added to RMR measurement cycles for the ambient treatments at Lower Deschutes and at North Umpqua to ensure a sufficient decrease in O_2_ for adequate MO_2_ measurements (Lower Deschutes: 1 minute added; North Umpqua: 4 minutes added). MMR was measured the following morning after 20 hours of RMR measurements. Fish were transferred into a bucket, chased by hand for 3 minutes, exposed to air for 1 minute (Little *et al.,* 2020a), and placed back into respirometers for 1 hour to measure MMR and exercise recovery. Most MMR measurements were taken within 1°C of the mean temperatures during the fluctuating acclimation treatments. MMR was measured within 2°C of mean holding temperatures for the Deschutes and John Day populations to meet our goal of obtaining aerobic scope at a challenging temperature for each population.

### Critical thermal maximum

After 1 hour of exercise recovery, CT_MAX_ tests were used to assess upper thermal tolerance. These tests were conducted immediately after respirometry when fish had been held at test temperatures for ~40 hours. Due to the propensity for rapid exercise recovery in juvenile *O. mykiss* (Dressler *et al.,* 2023) and that CT_MAX_ has often been found to be uninfluenced by aerobic stress (Ern *et al.,* 2023), we were not concerned that the fish had recently undergone a chase treatment, although prior acute stressors can influence CT_MAX_ (Rodgers and Gomez Isaza, 2022). CT_MAX_ start temperatures were always within 2°C of the chase temperature for each treatment (Table 2). To perform the CT_MAX_ test, fish were first placed into an aerated cooler and given 10 minutes to adjust to their surroundings. Then, water temperature was increased at a rate of 0.3°C min^−1^ (Beitinger *et al.,* 2000) by pumping heated water through a stainless-steel coil and dipping the coil in and out of the water. The temperature at loss of equilibrium (CT_MAX_) was recorded for each individual fish. When fish lost equilibrium, they were immediately netted and placed into an aerated bucket to recover. Fish were slowly brought back down to ambient stream temperatures and were released back into the wild.

**Table 2:** Oxygen uptake rates (MMR, RMR) at chase temperatures; AAS, FAS and CT_MAX_ for each temperature treatment and population

Population	Body size range (g)	Holding temperature fluctuation (°C)	Chase temperature (°C)	MMR (mg O_2_ kg^−1^ L^−1^)	RMR (mg O_2_ kg^−1^ L^−1^)	AAS (mg O_2_ kg^−1^ L^−1^)	FAS	CT_MAX_ start temperature (°C)	CT_MAX_ (°C)
Lower Deschutes	6.0–48.4	18–22	19	10.62 ± 0.54^a^	2.85 ± 0.12^a^	7.78 ± 0.61	3.76 ± 0.25^a^	20	32.06 ± 0.08^a^
		20–24	22	12.72 ± 0.29^b^	3.88 ± 0.24^b^	8.84 ± 0.29	3.34 ± 0.15^ab^	22	31.43 ± 0.06^b^
		23–27	26	12.59 ± 0.56^b^	4.60 ± 0.21^b^	7.99 ± 0.47	2.76 ± 0.12^b^	26	31.31 ± 0.11^b^
John Day	21.3–78.9	14–27	19	12.85 ± 0.89	2.42 ± 0.10^d^	10.42 ± 0.91^d^	5.42 ± 0.47^d^	21	31.22 ± 0.07
		20–24	22	15.23 ± 0.99	4.13 ± 0.28^d^	11.09 ± 0.83^d^	3.72 ± 0.20^e^	n/a	Not measured
		23–27	27	12.89 ± 1.74	6.56 ± 1.08^e^	6.32 ± 1.11^e^	2.21 ± 0.33^f^	26	31.16 ± 0.08
Siletz	15.1–44.2	15–19	16	12.27 ± 0.55	1.72 ± 0.09^g^	10.55 ± 0.50^g^	7.17 ± 0.36^g^	16	28.84 ± 0.09^g^
		18–22	19	13.86 ± 0.66	2.72 ± 0.23^h^	11.14 ± 0.49^g^	5.25 ± 0.23^gh^	20	29.50 ± 0.11^h^
		20–24	22	12.28 ± 1.45	3.19 ± 0.28^h^	9.10 ± 1.49^g^	4.02 ± 0.48^h^	22	30.24 ± 0.09^i^
		23–26	25	11.06 ± 0.52	4.70 ± 0.26^i^	6.36 ± 0.33^h^	2.36 ± 0.07^i^	n/a	Not measured
North Umpqua	9.7–42.1	16–19	16	9.76 ± 0.46	1.94 ± 0.09^j^	7.82 ± 0.49^j^	5.17 ± 0.37^j^	18	30.04 ± 0.33
		18–22	19	10.20 ± 0.47	2.37 ± 0.05^j^	7.83 ± 0.48^j^	4.33 ± 0.22^j^	19	29.88 ± 0.25
		20–24	22	10.15 ± 0.64	3.08 ± 0.09^k^	7.07 ± 0.64^jk^	3.31 ± 0.21^k^	22	30.03 ± 0.25
		23–26	25	9.54 ± 0.38	3.84 ± 0.18^l^	5.70 ± 0.35^k^	2.52 ± 0.11^k^	25	29.91 ± 0.38

All values are presented as mean ± SEM. All metabolic rate data are scaled to a common body size of 25 g. Differing letters indicate statistically significant differences between temperature treatments within populations (one-way ANOVA or Kruskal–Wallis test; *P* < 0.05; Deschutes River: a,b; John Day River: d,e,f; Siletz River: g,h,i; North Umpqua River: j,k,l).

### Data and statistical analysis

All data analysis was conducted in R version 4.2.2 with a significance level of α = 0.05 for statistical tests. Raw respirometry data were plotted and inspected for linearity both visually and by fitting linear regressions to the decline in dissolved oxygen over each measurement cycle. Measurement cycles with regressions with R^2^ < 0.9 and with clear data anomalies (i.e. patterns related to equipment rather fish oxygen consumption) were discarded. MO_2_ values were then obtained from each measurement cycle using the following equation: MO_2_ = (slope * (v_R_ − m))/m * (m/0.025)^(1 − scaling exponent)^, where v_R_ is the respirometer volume and m is the fish body weight in kilograms (R package: AnalyzeResp). Scaling exponents (0.74 for MMR and 0.72 for RMR) were obtained from linear regressions fitted to the log–log relationship between body mass and raw MO_2_ values across all populations and temperatures and including two additional steelhead populations from California (Dressler *et al.,* 2023; Fig. S1). All metabolic rate data were scaled to a common body mass of 25 g, the average body mass for all fish tested in this study, using these data-generated scaling exponents. Fish with more than 25% of MO_2_ regressions with R^2^ < 0.9 were excluded entirely from RMR analysis (<5% of fish across the entire study).

To calculate RMR, the first 240 minutes of data was discarded for each fish to ensure that they had recovered from handling stress. This cutoff time was determined visually from the data as the point where MO_2_ had fully settled for all fish posthandling. The mean temperature during each MO_2_ measurement was then rounded to the nearest degree, and MO_2_ values were averaged at each temperature during the diurnal fluctuation to represent the RMR at those temperatures. RMR calculations composed of *n* < 3 MO_2_ measurements for an individual fish were not included in statistical analysis. Due to fluctuating temperature treatments, we obtained RMR measurements from four to five temperatures for each fish, with the exception of the John Day ambient treatment, where we obtained RMR from 14 different temperatures. The effect of temperature on RMR and ln(RMR) was determined for each population with linear mixed models using both temperature and treatment as fixed effects and fish ID as a random effect (R package: lme4; Bates *et al.,* 2015). Results were obtained using Type II and Type III ANOVAs (R package: ‘car’; Fox and Weisberg, 2018), and Bayesian Information Criterion (BIC) was used to determine the best fit models.

To calculate MMR, we used the steepest 120-second slope from the measurement cycle where MMR occurred using a sliding window analysis (Little *et al.,* 2020a). In most cases, this was the first measurement cycle postchase, except for four fish where MMR occurred later in the recovery process. MMR always occurred postchase and never during RMR trials. MMR was compared between temperatures within populations using one-way ANOVAs.

Aerobic scopes were calculated using the RMR value that corresponded with the chase temperature (RMR_chase_). In other words, both MMR and RMR_chase_ were measured at the same temperature. AAS was calculated by subtracting RMR_chase_ from MMR. FAS was calculated by dividing MMR by the RMR_chase_. Both AAS and FAS were compared between treatments within populations using one-way ANOVAs. Quadratic polynomial functions were fit to the relationship between AAS and temperature, and regression analysis was used to determine the best fit. Optimal AAS temperatures (T_OPT_) as well as pejus temperatures (T_PEJ_) were calculated from these curves. T_OPT_ represents the temperature corresponding with the highest AAS, and T_PEJ_ represents the range of temperatures where fish have at least 80% of their peak AAS available to them (Clark *et al.,* 2013; Farrell, 2016). Linear regressions were fit to assess the relationship of FAS and temperature to determine the temperature, where FAS = 3 (T_FAS3_) for each population. FWT was calculated for each population by subtracting the T_FAS3_ from the maximum measured habitat temperature.

To assess the impact of temperature on exercise recovery, we examined how temperature influences the time it takes for each of our study populations to recover to a rate of oxygen consumption where they have 80% of their AAS available to them (Time_AAS80_) and until they have an FAS of 3 available to them (Time_FAS3_). Time_AAS80_ and Time_FAS3_ serve as additional metrics of functional thermal tolerance. MO_2_ was measured every ~10 minutes for 50–60 minutes after fish were chased. Biexponential decay models were fit to describe the decrease in MO_2_ over time for each treatment and temperature (Scarabello *et al.,* 1991). These models had the following formula: MO_2_(t) = Ae^αt^ + Be^βt^ + RMR, where t is time, α and A are the slope and y-intercept, respectively, of the first exponential decay, β and B are the slope and y-intercept, respectively, of the second exponential decay, and RMR is the average RMR_chase_ for each corresponding population and temperature. These models describe the average decay of MO_2_ over time for each population and temperature. To solve for Time_AAS80_ and Time_FAS3_, we used the RMR_chase_ of each individual fish in these models and found the time point (rounded to the nearest 0.1 second) where MO_2_ was equal to 80% of the fish’s AAS (for Time_AAS80_) and where MMR divided by MO_2_ was equal to 3 (i.e. time to recovery to FAS = 3, Time_FAS3_).

CT_MAX_ was compared between temperature treatments using one-way ANOVAs, except for the John Day population where treatments were compared using a student’s *t* test (CT_MAX_ was only measured for two of the three treatments at this site). CT_MAX_ was compared between populations at common temperature treatments using Kruskal–Wallis tests. TSMs were calculated for each population by subtracting the average CT_MAX_ at ambient temperatures from the maximum measured stream temperature.

## Results

### Habitat temperature characteristics

Habitat temperatures indicate that all four summer steelhead populations experience distinct thermal regimes in their respective habitats (Table 1 and Fig. 1). The John Day and Lower Deschutes reached the warmest temperatures during the summer months with maximum temperatures of 24.9°C (July 2022) and 27.1°C (July 2021), respectively. North Umpqua reached intermediate temperatures, with a maximum summer temperature of 21.6°C (July 2022). Siletz remained the coolest during the summer with a maximum temperature of 15.9°C (June 2021). Daily variability during the summer months (June, July and August) ranged from 3°C to 13°C at John Day, 1°C to 7°C at Lower Deschutes, 1°C to 6°C at North Umpqua and 0.4°C to 4°C at Siletz. All temperatures approached freezing during the winter months, but exact temperature minimums are uncertain due to our data loggers having unreliable readings at temperatures <4°C.

### Critical thermal maximum

CT_MAX_ ranged from 27.4°C to 32.5°C and varied in magnitude and plasticity across summer steelhead populations (Table 2). *O. mykiss* from the Siletz had increasing CT_MAX_ with increasing holding temperatures (ANOVA, *P* < 0.001), while *O. mykiss* from the North Umpqua and John Day showed no change in CT_MAX_ with increasing holding temperatures (ANOVA, *P* = 0.967, and t-test, *P* = 0.605, respectively). Lower Deschutes *O. mykiss* had slightly decreased CT_MAX_ at temperatures above ambient (ANOVA, *P* < 0.001). At common holding temperature treatments of 18–22°C and 20–24°C, the Lower Deschutes population had significantly higher CT_MAX_ than the Siletz and North Umpqua populations (Kruskal–Wallis tests, *P* < 0.001 for both 19°C and 22°C). At a common trial starting temperature of 19°C, CT_MAX_ of the John Day population was higher than the North Umpqua and Siletz populations but lower than the Lower Deschutes population, with the caveat that during this treatment, the John Day population experienced a much wider range of temperatures (14–27°C) compared to the other populations (18–22°C). TSMs varied between populations and ranged from 4.1°C to 12.9°C.

### Metabolic rate

**Figure 4:**
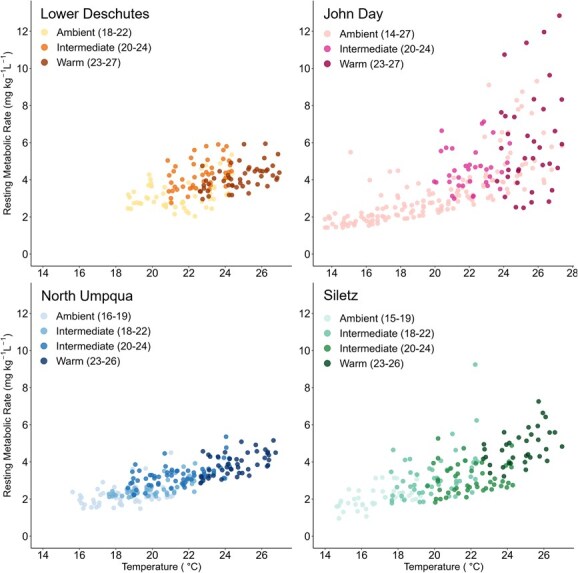
RMR for all treatments and populations. Points indicate mean RMR for individual fish at each temperature, and points have been jittered to easily discern holding temperature treatments from one another. Points are shaded to differentiate holding temperature treatments, with ambient treatments as the lightest colours and the warmest treatments as the darkest colours.

RMR increased exponentially with temperature for all populations and was influenced by both acute temperatures during the diurnal fluctuations and by holding temperature treatments (Fig. 4). For the Lower Deschutes and North Umpqua populations, there were significant effects of acute temperature and holding temperature treatment, but not their interaction, on RMR (Table S2). Siletz *O. mykiss* showed significant effects of acute temperature, treatment and their interaction on RMR (Table S2). For the John Day population, there were significant effects of acute temperature and the interaction between acute temperature and holding temperature treatment but no effect of treatment itself (Table S2). At the common holding temperature treatment of 20–24°C, John Day and Lower Deschutes *O. mykiss* had 20–50% higher RMR at all temperatures compared to Siletz and North Umpqua *O. mykiss* (Fig. S3).

**Figure 5:**
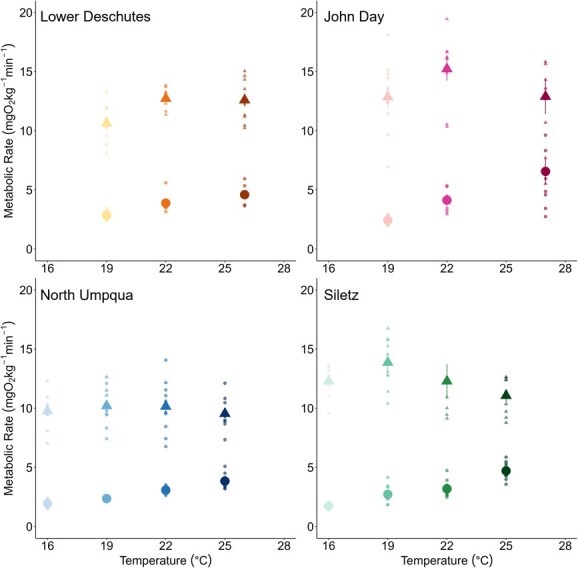
MMR (triangles) and RMR (circles) for all populations at each of the chase temperatures. Large filled triangles indicate mean ± SEM MMR, and large filled circles indicate mean ± SEM RMR at each temperature. Small triangles represent MMR measurements from individual fish, and small circles represent RMR measurements from individual fish at each of the chase temperatures. Colour shading indicates holding temperature treatment with ambient treatments as the lightest colours and the warmest treatments as the darkest colours.

**Figure 6:**
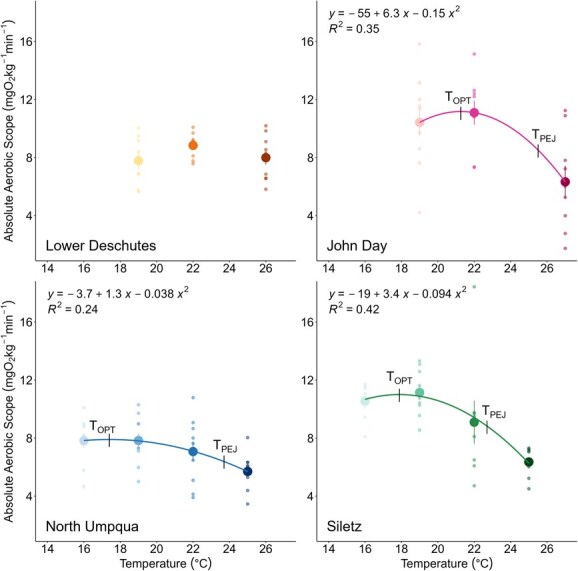
AAS for all populations. Small transparent points represent AAS measurements for individual fish. Large filled points indicate mean ± SEM AAS at each temperature. Curves and equations represent quadratic polynomial functions fitted to describe the relationship between AAS and temperature. T_OPT_ and T_PEJ_ are denoted on each curve.

**Figure 7:**
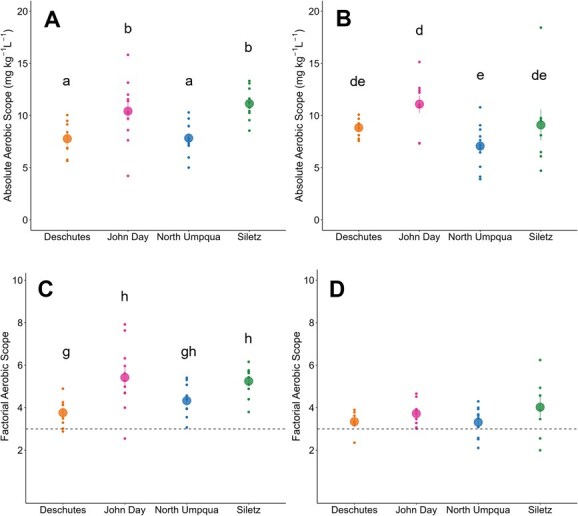
AAS (**A** and **B**) and FAS (**C** and **D**) for the Lower Deschutes, John Day, North Umpqua, and Siletz populations tested at common temperatures (left: 19°C; right: 22°C). Small points indicate AAS or FAS of individual fish, and large points indicate mean ± SEM AAS or FAS for each population. Lowercase letters indicate statistically significant differences between populations (ANOVA, *P* < 0.05).

Overall, steelhead MMR was not strongly affected by test temperatures (Table 2 and Fig. 5). MMR did not change with temperature for John Day, North Umpqua and Siletz populations (ANOVAs, *P* = 0.185, 0.721 and 0.125, respectively). For the Lower Deschutes population, MMR increased between 19°C and 22°C but did not differ between 22°C and 26°C (ANOVA, *P* = 0.009). The effect of temperature on AAS, however, was population dependent. AAS did not change between test temperatures for the Lower Deschutes population (Table 1 and Fig. 6, ANOVA, *P* = 0.261). Polynomial curves were fit to the relationship between AAS and temperature for the John Day, North Umpqua and Siletz populations. T_OPT_ ranged from 17°C to 21°C and T_PEJ_ between 21°C and 23°C for these three populations, and in all cases, there was a significantly lower AAS at the warmest test temperature (Table 2 and Fig. 6). AAS also varied between populations when tested at common temperatures. The Siletz and North Umpqua populations were tested at all the same temperatures, and the Siletz population had a higher AAS at all temperatures except 25°C, where AAS was not significantly different. At 19°C, the John Day and Siletz populations had a higher AAS compared to the Lower Deschutes and North Umpqua populations (Fig. 7). At 22°C, the John Day population had a significantly higher AAS than the North Umpqua population, but all other pairwise AAS comparisons were not significantly different (Fig. 7).

**Figure 8:**
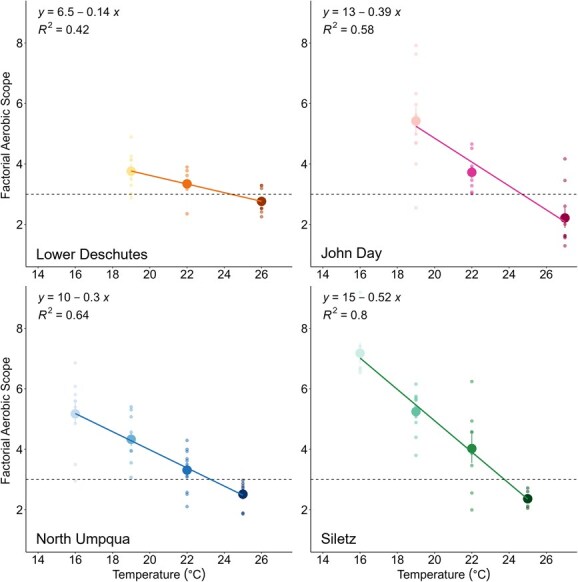
FAS for all populations. Small transparent points represent FAS measurements for individual fish. Large filled points indicate mean ± SEM FAS at each chase temperature. Solid lines and equations represent linear models fitted to describe the relationship between FAS and temperature. Dashed lines indicate FAS = 3 (T_FAS3_; Lower Deschutes: 24.4°C, John Day: 24.9°C, North Umpqua: 23.3°C, Siletz: 23.8°C).

FAS decreased linearly with increasing temperatures for all populations (Table 2 and Fig. 8). Model selection confirmed that the best fit included a unique slope and y-intercept for each population rather than an average of all four populations. The regression for the Lower Deschutes population is shallower (slope = 0.14) compared to the others (slopes = 0.3–0.46), meaning that FAS for this population was less temperature sensitive compared to the others, at least across the temperatures tested herein. T_FAS3_ temperatures ranged from 23.8°C to 24.9°C, and FWT varied widely, ranging from −2.2°C to 7.9°C (Table 2).

### Exercise recovery

**Figure 9:**
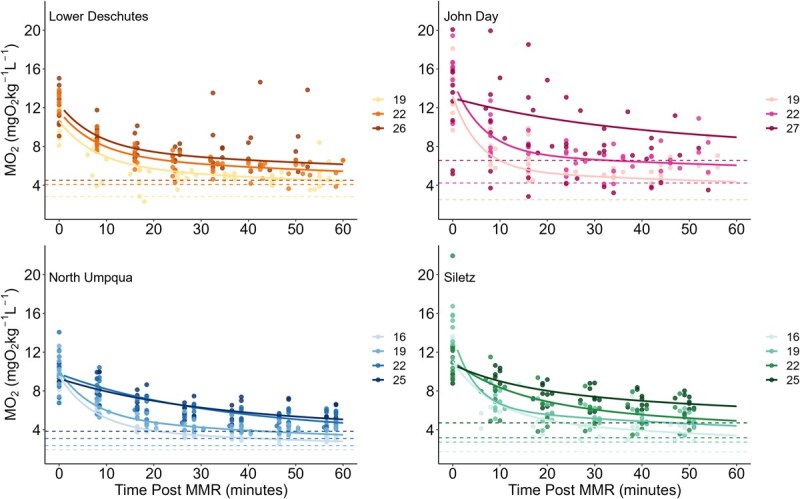
Oxygen uptake rate (MO_2_) over time for each population and chase temperature during 1 hour of exercise recovery postchase. Circular data points represent MO_2_ measurements for individual fish with MMR at x = 0. Solid curve lines show the predicted MO_2_ values based on biexponential decay functions fit to the relationship between MO_2_ and time postchase for each population and temperature. Dashed horizontal lines indicate RMR for each population and temperature.

**Figure 10:**
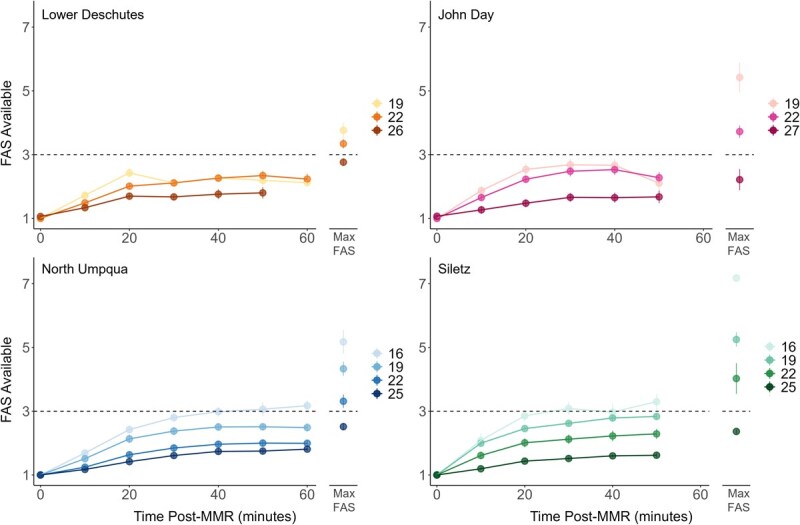
FAS available to the fish (i.e. MMR divided by MO_2_) over time for 1 hour of postchase exercise recovery. Points represent mean ± SEM FAS available for each population and temperature every 10 minutes postchase. The point at which each recovery profile crosses the dashed horizontal line represents the time that an FAS of 3 is available to the fish at each temperature. The mean ± SEM FAS (i.e. MMR divided by resting MO_2_ [RMR]) measured at each temperature is shown to the right of each recovery profile to give perspective on how much aerobic scope is recovered after 1 hour postchase.

After MMR, MO_2_ decreased in a biexponential decay pattern for the entirety of the 50- to 60-minute recovery period. The first, steeper exponential decay occurred between time 0–20 min after MMR and the second, shallower exponential decay occurred between 20-60 min after MMR (Fig. 9). Temperature had a significant impact on recovery timing, with higher temperatures resulting in prolonged recovery (higher Time_AAS80_ and Time_FAS3_) for the John Day, Siletz and North Umpqua populations (Figs 9 and 10, Table 3). For the John Day population, recovery was impaired between 22°C and 27°C (Figs 9 and 10, Table 3). For the Siletz and North Umpqua populations, recovery was impaired between 19°C and 22°C (Figs 8 and 9, Table 3). Temperature did not impact recovery timing for the Lower Deschutes population, but Time _FAS3_ is significantly higher than the other populations (Table 3). At common temperatures of 19°C and 22°C, the John Day population had a significantly lower Time_AAS80_ and Time_FAS3_ compared to the other populations and therefore had the fastest exercise recovery (Table 3). The Lower Deschutes population had the highest T_FAS3_ (and therefore the slowest recovery of FAS) at 19°C and a higher Time_FAS3_ than the John Day and Siletz populations at 22°C.

**Table 3:** Exercise recovery metrics for each temperature treatment and population including the time to have 80% of AAS available (Time_AAS80_) and the time to have an FAS of 3 available (Time_FAS3_) after being chased

Population	Holding temperature fluctuation (°C)	Chase temperature (°C)	Time_AAS80_ (minutes)	Time_FAS3_ (minutes)
Lower Deschutes	18–22	19	71.0 ± 13.6^a^	162.1 ± 0.12^a^
	20–24	22	50.0 ± 2.6^a^	156.6 ± 0.24^a^
	23–27	26	60.0 ± 4.9^a^	n/a
John Day	14–27	19	11.2 ± 0.5^d^	12.1 ± 1.0^d^
	20–24	22	12.1 ± 0.7^d^	24.5 ± 4.3^e^
	23–27	27	34.1 ± 4.3^e^	n/a
Siletz	15–19	16	41.5 ± 3.9^g^	39.0 ± 4.5^g^
	18–22	19	37.7 ± 3.5^g^	51.7 ± 6.1^gh^
	20–24	22	66.2 ± 9.2^h^	88.7 ± 21.3^h^
	23–26	25	83.5 ± 4.4^h^	n/a
North Umpqua	16–19	16	29.2 ± 3.3^j^	36.0 ± 8.8^j^
	18–22	19	40.7 ± 3.4^j^	77.5 ± 16.6^j^
	20–24	22	66.4 ± 4.5^k^	125.8 ± 19.9^k^
	23–26	25	64.2 ± 3.1^k^	n/a

All values are presented as mean ± SEM. Differing letters indicate statistically significant differences between temperature treatments within populations (one-way ANOVA or Kruskal–Wallis test; *P* < 0.05; Deschutes River: a,b; John Day River: d,e,f; Siletz River: g,h,i; North Umpqua River: j,k,l).

## Discussion

Here we measured aerobic scope, exercise recovery and CT_MAX_ of four populations of juvenile summer-run steelhead trout exposed to acute, ecologically relevant temperature increases. We found clear intraspecific differences in thermal performance across populations. As predicted, the thermal tolerance of this species varies across a gradient of habitat temperature conditions rather than latitude, highlighting the need for population-specific management strategies for this species and ecotype. While we cannot identify the mechanism underlying these intraspecific differences (i.e. whether these differences were due to long-term thermal acclimatization, parental effects and/or local adaptation), it is clear that the populations currently experiencing the warmest temperatures are living close to their thermal limits and are likely to face physiological challenges if temperatures continue to increase.

### TSMs differed across populations

CT_MAX_ values were all within the range previously measured for *O. mykiss* (24–32°C; Zhang *et al.,* 2018, Recsetar *et al.,* 2012, reviewed in McKenzie *et al.,* 2021), and the populations from the warmest locations, John Day and Lower Deschutes, were at the upper end of this range (i.e. 30.3–32.5°C). As expected, the John Day and Lower Deschutes populations had higher CT_MAX_ than the Siletz and North Umpqua (cooler locations) populations and most other previously studied *O. mykiss* with the exception of two warm-adapted hatchery strains in Western Australia and Arizona, USA, and a wild population at the southern end of the species’ native range in California, USA (Table 2; Recsetar *et al.,* 2012; Adams *et al.,* 2022; Dressler *et al.,* 2023). The John Day population experienced ambient temperature swings of 14–27°C during experiments, and CT_MAX_ of fish exposed to this swing and tested at 19°C was the same as fish exposed to just the upper end of this swing (23–27°C) and tested at 27°C. In this case, CT_MAX_ showed limited plasticity and appears to be associated with the warm end of this diurnal temperature swing. The other warm-acclimatized population (Lower Deschutes) displayed a similarly high CT_MAX_ overall, but no improvement when exposed to higher temperatures. In contrast, the population from the coldest habitat, Siletz, had the lowest CT_MAX_ in ambient conditions, but CT_MAX_ displayed rapid plasticity, increasing with acclimation exposure to warmer temperatures (Table 2). Results from the John Day, Siletz and Lower Deschutes populations provide evidence of an acclimation ceiling for CT_MAX_, meaning that upper thermal limits of warm-dwelling summer steelhead are unlikely to be able to acclimate if temperatures continue to increase (Sandblom *et al.,* 2016). The North Umpqua population, however, had a lower CT_MAX_ than the John Day and Lower Deschutes populations at common temperatures, and CT_MAX_ did not exhibit rapid acclimation (Table 2). This population also had more interindividual variability than any of the other populations (Table 2). It could be that this population takes longer than 40 hours to start acclimating, and the interindividual variability is an artefact of some individuals beginning to acclimate faster than others. Another possible explanation is that the North Umpqua population relies more on local adaptation than phenotypic plasticity for adjusting their upper thermal limits in response to warming. Regardless, managers should be aware that while summer steelhead in the North Umpqua may have the capacity to increase their upper thermal limits, they are not able to do so over a rapid timescale characteristic of heat waves in this area.

While the Lower Deschutes and John Day populations had high critical thermal limits, they also had lower TSMs compared to the North Umpqua and Siletz populations. Notably, the John Day population had the lowest TSM of 4.1°C, meaning that ambient temperatures would only have to increase by ~4°C for the John Day population to reach its lethal limits (and it is unlikely these fish would be able to acclimate given the fast rate of temperature change in this system and the observed lack of plasticity of CT_MAX_). By comparison, the other three populations have more substantial buffers between their lethal limits and current maximum habitat temperatures (Table 4). While a low TSM may alert managers that a given population is at imminent risk of extirpation, moderate and high TSMs have limited utility to managers because temperature limits fish physiological function below CT_MAX_ temperatures.

**Table 4:** Thermal vulnerability metrics for each *O. mykiss* population including thermal safety margins (TSM) and functional warming tolerance (FWT). Also included are optimal (T_OPT_) and pejus (T_PEJ_) temperatures for AAS, temperatures where FAS = 3 (T_FAS3_), average critical maxima (CT_MAX_) at ambient holding temperatures, and maximum stream temperatures measured during this study.

Watershed	T_OPT_	T_PEJ_	T_FAS3_	CT_MAX_	Max stream temperature	TSM	FWT
Lower Deschutes	–	–	24.4	32.1	24.9	7.2	−0.5
John Day	21.3	17.4, 25.2	24.9	31.2	27.1	4.1	−2.2
North Umpqua	17.4	11.0, 23.7	23.3	30.0	21.6	8.4	1.7
Siletz	17.9	13.0, 22.7	23.8	28.8	15.9	12.9	7.9

All values are in °C.

### Energetic costs and FWT differed across populations

Based on differences in RMR between populations at a common temperature treatment of 20–24°C, the populations that experience warmer summer temperatures (Lower Deschutes, John Day) likely need to eat more to keep up with their maintenance metabolic costs. The Lower Deschutes and John Day population had 20–50% higher RMR than the North Umpqua and Siletz populations. This result is uncommon, as prolonged warm exposure tends to result in reduced RMR (Healy and Schulte, 2012; Mcbryan *et al.,* 2016; Sandblom *et al.,* 2016; Railsback, 2022) but is consistent with a similar study on *O. mykiss* populations in California (Dressler *et al.,* 2023). We cannot be certain that these differences in RMR are consistent across all temperatures, but it is noteworthy that the North Umpqua and Siletz populations have a lower RMR at their T_PEJ_ and T_FAS3_, which fall within the range of this 20–24°C common temperature treatment (Table 4). A high RMR indicates that the fish have higher costs for maintenance metabolism, and thus a greater amount of the energy consumed by these fish is allocated first to ensure basic baseline function before excess energy can be allocated to fitness-enhancing performances such as swimming and digestion. While it is notable that *O. mykiss* populations from warm environments tend to have higher baseline oxygen requirements, it is also possible that factors other than temperature such as disease (Powell *et al.,* 2005; Ogut and Parlak, 2014) and growth rate (Greenaway *et al.,* 2024) contribute to this discrepancy.

The interpopulation variation in the thermal performance curve for AAS reveals a tradeoff between magnitude and thermal sensitivity of aerobic scope that seems to be associated with habitat temperature regimes. As habitat temperature gets warmer, summer steelhead populations appear to sacrifice the magnitude of peak AAS in favour of a broad T_OPT_ window for AAS. The Siletz population experiences the coolest temperatures (Fig. 1) and has a 38% higher peak AAS and a 24% narrower T_OPT_ window compared to the North Umpqua population (Fig. 6) that experiences intermediate temperatures (Fig. 1). The Lower Deschutes population experiences high temperatures (Fig. 1) and displayed a low AAS and an extremely broad T_OPT_ window, such that this population had the same AAS at 19°C, 22°C and 26°C, similar to a southern California population in Dressler *et al.* (2023). At 26°C, the Lower Deschutes population has a higher AAS compared to the Siletz and North Umpqua populations at 25°C, demonstrating the payoff of having a reduced thermal sensitivity. However, reduced AAS at more intermediate temperatures indicates that this population is likely to have a reduced capacity for functions like growth and predator evasion.

John Day summer steelhead were the exception to this trend of trading off peak AAS with thermal breadth. However, this population inhabits a stream that has unique thermal characteristics compared to the others. This stream reaches the warmest peak temperatures of all our study locations but was also the most variable, fluctuating by up to 13°C daily. This population has a similar peak AAS (11.14 mg O_2_ kg^−1^ L^−1^) as the Siletz population and a slightly narrower T_OPT_ window (7.8°C). The AAS curve for this population was right shifted and therefore had a higher T_OPT_ and upper T_PEJ_ compared to the Siletz and North Umpqua populations. Daily variability of habitat temperatures can therefore also lead to population differences in thermal tolerance. While the John Day and Lower Deschutes populations both experience warm maximum summer temperatures, it is possible that the John Day population does not invest in acclimation to these temperatures because they only occur briefly during the day. It is also worth noting that population differences in AAS can also be related to other selective factors including migration distance, flow rates and gradient and presence of predators or competitors (e.g. Sloman *et al.,* 2000; Millidine *et al.,* 2006; Eliason *et al.,* 2011). John Day summer steelhead have the longest migration of the four populations (Table 1), and Siletz summer steelhead compete with coastal cutthroat trout in the tributary where we obtained the fish. These factors may contribute to these two populations having higher peak AAS than the others.

As hypothesized, the increased thermal tolerance of the populations from warm habitats was not enough to confer a substantial buffer to warming. FAS was least temperature sensitive (i.e. slope of the decline was shallowest) for the Lower Deschutes, and Time_AAS80_ did not change between 19°C and 26°C, reaffirming that this population is the least temperature sensitive. T_FAS3_ did not vary as much as expected (23–25°C), but FWT varied greatly between populations. John Day summer steelhead had the highest T_FAS3_, and recovery was not prolonged until 27°C but had the lowest FWT of −2.2°C, indicating that current temperatures exceed the functional thermal limits for these fish. Lower Deschutes had an FWT of −0.5, indicating that current maximum habitat temperatures reach the functional thermal limits for this population. The North Umpqua population has a slight buffer against warming (FWT = 1.7°C), but recovery was impaired at 22°C (0.4°C from the maximum measured stream temperature), suggesting that this population may soon experience physiological limitations from temperature. One caveat is that we do not have information on spatial thermal heterogeneity in these tributaries, and therefore cannot be sure whether thermal refugia are available to these fish. Of the study populations, the North Umpqua fish are most likely to have access to thermal refugia as the landform, vegetation structure and heavy precipitation in this system have given way to deep pools. The Lower Deschutes and John Day habitats are comparatively narrow and shallow, and fish are more likely to have to rely on overnight cooling as a source of thermal refugia. In any case, projected decreases in streamflow and increases in water temperatures are predicted to cause existing thermal refugia to shrink and not support as many individuals in the near future (Mantua *et al.,* 2010). The Siletz population had the largest FWT of 7.9°C, a substantial buffer against warming. While coastal summer steelhead populations such as the Siletz should still be monitored to track trends in temperature, it is unlikely that temperature will be a physiological limitation for these fish. In contrast, inland populations will require more active management efforts as well as further studies linking physiology with trends in behaviour and food resources (e.g. Hahlbeck *et al.,* 2023).

### Exercise recovery timing varied between populations and metrics

**Figure 11:**
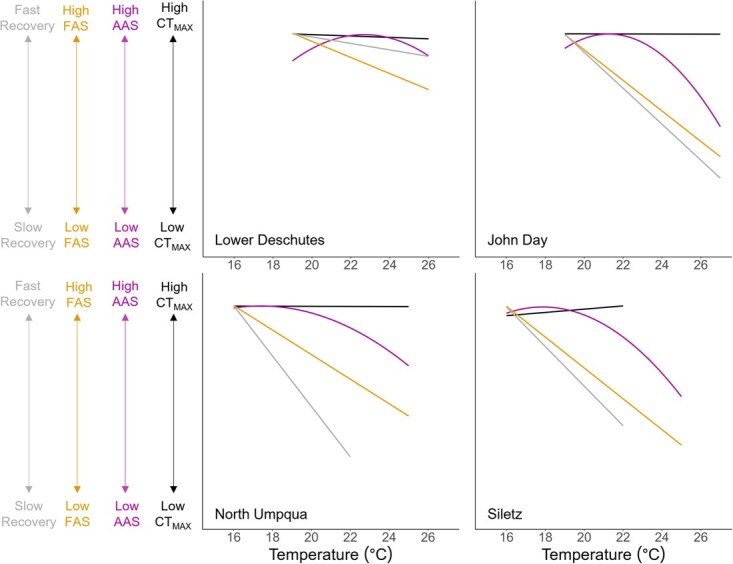
Summary of the relative response of critical thermal maximum (CT_MAX_), AAS, FAS and exercise recovery (Time_FAS3_; recovery) to increasing temperature. All curves are shown as a percentage of the maximum measured value for the corresponding population.

Here, we quantified exercise recovery using two novel metrics (Time_AAS80_ and Time_FAS3_) to approximate the time it took each fish to reach a level of recovery where they could resume normal activities. Exercise recovery is often quantified using a three-phase curve fit between the time of MMR and the time that standard metabolic rate (SMR) is reached. The area under the curve is used to calculate the amount of oxygen consumed by the fish during the recovery period (excess postexercise oxygen consumption; Zhang *et al.,* 2018). It can take up to 12 hours for a fish to fully recover to SMR, during which metabolites and stress hormones are restored to baseline levels (Scarabello *et al.,* 1991; Lee *et al.,* 2003; MacNutt *et al.,* 2006; Eliason *et al.,* 2013; Zhang *et al.,* 2018). However, salmonids can resume aerobically challenging activities after partial recovery (Farrell *et al.,* 1998; Lee *et al.,* 2003; MacNutt *et al.,* 2006; Eliason *et al.,* 2013; Eliason and Farrell, 2016), and fish are unlikely to have multiple hours to rest and recover in the wild. We opted instead to measure recovery over 1 hour to capture the initial phase of rapid recovery and part of the plateau phase. In general, summer steelhead took longer to reach Time_FAS3_ compared to Time_AAS80_ at temperatures of 19°C and above (Table 3). Since FAS ≥3 is needed for feeding and digestion, this metric is likely more relevant to this juvenile life stage than Time_AAS80_, which may be more relevant for migratory life stages (Eliason *et al.,* 2023). Of all the performances measured in this study, exercise Time_FAS3_ was typically the most sensitive to temperature increase, followed by FAS, AAS and CT_MAX_, respectively (Fig. 11). In other words, the ability to recover from fisheries and predator interactions is the first function to become impaired by temperature in juvenile summer steelhead, a pattern that has also been observed in adult coho salmon (Kraskura *et al.*, 2020). Time_FAS3_ was significantly higher for the Lower Deschutes population, even at nonstressful temperatures, than any of the other populations, meaning that while this population is more resistant to incurring higher energetic costs at warm temperatures, costs of recovering from aerobic efforts are high at all tested temperatures. This means that Lower Deschutes summer steelhead could be more susceptible to mortality from predator evasion or catch-and-release fishing. In contrast, the John Day population recovered extremely quickly at nonstressful temperatures (Table 3). This is likely advantageous given the large daily temperature swings these fish encounter and could be a result of local adaptation to this variable environment. Energetic costs incurred during the brief time that temperatures are hot can likely be quickly recuperated once temperatures start to cool.

## Conclusions

The present study documents intraspecific differences in thermal tolerance between populations of summer steelhead inhabiting distinct thermal environments located within Oregon, USA. While historically warm habitat conditions appear to confer elevated functional and critical thermal tolerance, this does not guarantee reduced vulnerability to climate warming. In fact, warm-dwelling summer steelhead populations appear to be at the greatest risk of experiencing physiologically limiting temperatures. Therefore, managers should focus on active conservation efforts such as habitat restoration on warm-dwelling, inland populations for this species. Population-specific management strategies, particularly for broadly distributed species like steelhead, will be crucial for mitigating the impact of climate change on fishes.

## Supplementary Material

Web_Material_coaf030

## Data Availability

Data are available on Dryad (https://doi.org/10.5061/dryad.brv15dvkp), and R code used for data analysis is available on GitHub (https://github.com/terradressler/2024_Dressler_et_al_Cons_Phys_OregonSST).
