# Supplementary material for: Beyond latitude: thermal tolerance and vulnerability of a broadly distributed salmonid across a habitat temperature gradient
Source: Conserv Physiol. 2025 May 1;13(1):coaf030. doi: 10.1093/conphys/coaf030 (PMC12043440; doi:10.1093/conphys/coaf030)
Supplement: Web_Material_coaf030 [file web_material_coaf030.zip › Dressler_et_al_Supplemental_Material_changes_accepted.pdf]

Supplemental Material

Table S1. Respirometry methods summary (adapted from Killen et al., 2021).

**Criterion and Category**	**Response**
**EQUIPMENT, MATERIALS, AND SETUP**	
Body mass of animals at time of respirometry	6-79 g
Volume of empty respirometers	2.1 L
How chamber mixing was achieved	Recirculation pump (Eheim Universal 300-L h^{-1})
Ratio of net respirometer volume (plus any associated tubing in mixing circuit) to animal body mass	84:1 (mean)
Material of tubing used in any mixing circuit	PVC
Confirm volume of tubing in any mixing circuit was included in calculations of oxygen uptake	confirmed
Material of respirometer (e.g., glass, acrylic, etc.)	plastic
Type of oxygen probe and data recording	FirestingO2
Sampling frequency of water dissolved oxygen	Every 1-2 seconds
Describe placement of oxygen probe (in mixing circuit or directly in chamber)	In mixing circuit
Confirm that chamber returned to normoxia during flushing	Confirmed
Timing of flush/closed cycles	6 minutes closed, 4 minutes flush
Wait (delay) time excluded from closed measurement cycles	30 seconds
Frequency and method of probe calibration (for both 0 and 100% calibrations)	Once at the beginning of each field excursion.
**MEASUREMENT CONDITIONS**	
Temperature during respirometry	Ranged from 14-27°C between treatments, fluctuated by 4-5°C within treatments (except for the John day ambient treatment).
How temperature was controlled	Pentair Smart One Easy Plug Axial Heaters
Photoperiod during respirometry	Natural (experiments conducted outside)
If (and how) ambient water bath was cleaned and aerated during measurement of oxygen uptake	Continuous flow of water in from the stream into a header tank. Header tank was equipped with electric aerators.
Total volume of header tank.	380 L

Minimum water oxygen dissolved oxygen reached during closed phases	80%
State whether chambers were visually shielded from external disturbance	Yes, respirometry tanks were situated under a shade tent.
Duration of animal fasting before placement in respirometer	~20 hours
Duration of all trials combined (number of days to measure all animals in the study)	15 days of respirometry
Acclimation time to the laboratory (or time since capture for field studies) before respirometry measurements	20 hours in holding tank
**BACKGROUND RESPIRATION**	
State whether background microbial respiration was measured and accounted for, and if so, method used	Background measured in an empty chamber throughout each trial.
State how changes in background respiration were modelled over time (e.g., linear, exponential, parallel measures)	Background was negligible, therefore not modelled
Level of background respiration (e.g., as a percentage of SMR)	0% SMR
**STANDARD OR ROUTINE METABOLIC RATE**	
Time period, within a trial, over which oxygen uptake was measured (e.g., number of hours)	20 hours of RMR ; 1 hour of MMR/Recovery
Value taken as SMR/RMR (e.g., quantile, mean of lowest 10 percent, mean of all values)	RMR: mean of all values at a given temperature
Whether any time periods were removed from calculations of SMR/RMR	First 240 minutes were removed from the beginning of each RMR trial to allow fish to reach a calm state.
r^2 threshold for slopes used for SMR/RMR	0.9
**MAXIMUM METABOLIC RATE**	
When MMR was measured in relation to SMR (i.e. before or after)	after
Method used	3 min chase, 1 min air exposure
Value taken as MMR (e.g., the highest rate of oxygen uptake value after transfer, average of highest values)	Steepest 120 s slope after transfer
If MMR measured post-exhaustion, time until transfer to chamber after exhaustion or time to start of oxygen uptake recording	< 30 s
Duration of slopes used to calculate MMR	Steepest 120 s slope from a 4-minute measurement slope
How absolute aerobic scope and/or factorial aerobic scope is calculated (i.e. using raw SMR and MMR, allometrically mass-adjusted SMR and MMR, or allometrically mass-adjusting aerobic scope itself)	Allometrically mass-adjusted RMR and MMR.

**DATA HANDLING AND STATISTICS**	
Sample size	
How oxygen uptake rates were calculated (software or script, equation, units, etc.)	Respirometry Performances R package
Confirm that volume (mass) of animal was subtracted from respirometer volume when calculating oxygen uptake rates	confirmed

Table S2: Model selection for relationships between resting metabolic rate (RMR), acute temperature, nd temperature treatment. Best fit models are highlighted in gray.

Population	***Dependent Variable***	***Fixed Effect***	X^2	***Df***	***p-value***	***BIC***
Lower Deschutes	RMR	Acute Temperature	26.7078	1	**<0.001**	269.41
		Treatment	6.9115	2	**0.032**	
		Acute Temperature: Treatment	10.3037	2	**0.006**	
	ln(RMR)	Acute Temperature	24.7388	1	**<0.001**	-131.77
		Treatment	2.6732	2	0.263	
		Acute Temperature: Treatment	4.9332	2	0.085	
	ln(RMR)	Acute Temperature	79.379	1	**<0.001**	-137.04
		Treatment	15.680	2	**<0.001**	
John Day	RMR	Acute Temperature	276.8700	1	**<0.001**	708.11
		Treatment	3.8573	2	0.145	
		Acute Temperature: Treatment	3.1066	2	0.212	
	RMR	Acute Temperature	274.0674	1	**<0.001**	700.41
		Treatment	1.6324	2	0.442	
	RMR	Acute Temperature	287.31	1	**<0.001**	691.22
	ln(RMR)	Acute Temperature	569.3172	1	**<0.001**	2.86
		Treatment	2.7799	2	0.249	
		Acute Temperature: Treatment	13.0620	2	**0.001**	
North Umpqua	RMR	Acute Temperature	22.6364	1	**<0.001**	295.05
		Treatment	5.7491	3	0.124	
		Acute Temperature: Treatment	6.5360	3	0.089	
	RMR	Acute Temperature	132.080	1	**<0.001**	285.35
		Treatment	16.844	3	**<0.001**	
	ln(RMR)	Acute Temperature	36.1010	1	**<0.001**	-188.08
		Treatment	5.4436	3	0.142	
		Acute Temperature: Treatment	3.2050	3	0.361	
	ln(RMR)	Acute Temperature	131.406	1	**<0.001**	-201.04
		Treatment	24.544	3	**<0.001**	
Siletz	RMR	Acute Temperature	11.3632	1	**<0.001**	425.51
		Treatment	18.3869	3	**<0.001**	
		Acute Temperature: Treatment	22.5578	3	**<0.001**	
	ln(RMR)	Acute Temperature	41.000	1	**<0.001**	-35.64
		Treatment	10.429	3	**0.015**	
		Acute Temperature: Treatment	12.633	3	**0.006**	

Figure S1: Log-log relationships between body weight and Maximum Metabolic Rate (MMR; panels A and B) and between body weight and Resting Metabolic Rate (RMR; panels C and D). In panels A and C, color indicates *O. mykiss* population. In panels B and D, shading represents the temperature at which each metabolic rate measurement was taken, with lighter shading indicated warmer temperaures and darker shading indicating cooler temperaures. The majority of these data are from the present study but data collected from 2 wild California *O. mykiss* populations (Dressler et al. 2023) have also been incoprated here. These data were collected using the same methodology and strengthen the dataset by increasing sample size at both ends of the size distribution.

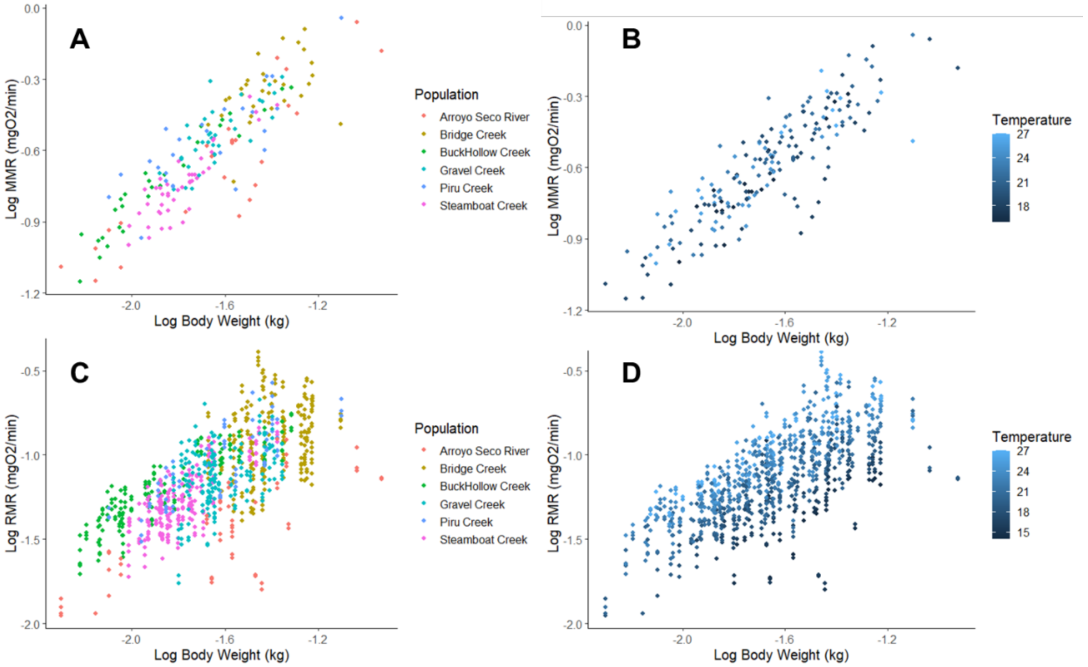

Figure S2. A representative trace of metabolic rate of a single fish over time (circular points) after it was transferred into a respirometer. The red shaded area shows the first 240 minutes that were excluded from Resting Metabolic Rate (RMR) calculations to ensure that fish had recovered from handling stress when RMR measurements began. The solid line shows the temperature in the respirometer over the duration of the RMR trial. Blue shading indicates temperature rounded to the nearest °C. Points with matching shading were averaged to determine the RMR of this fish at each of the rounded temperatures.

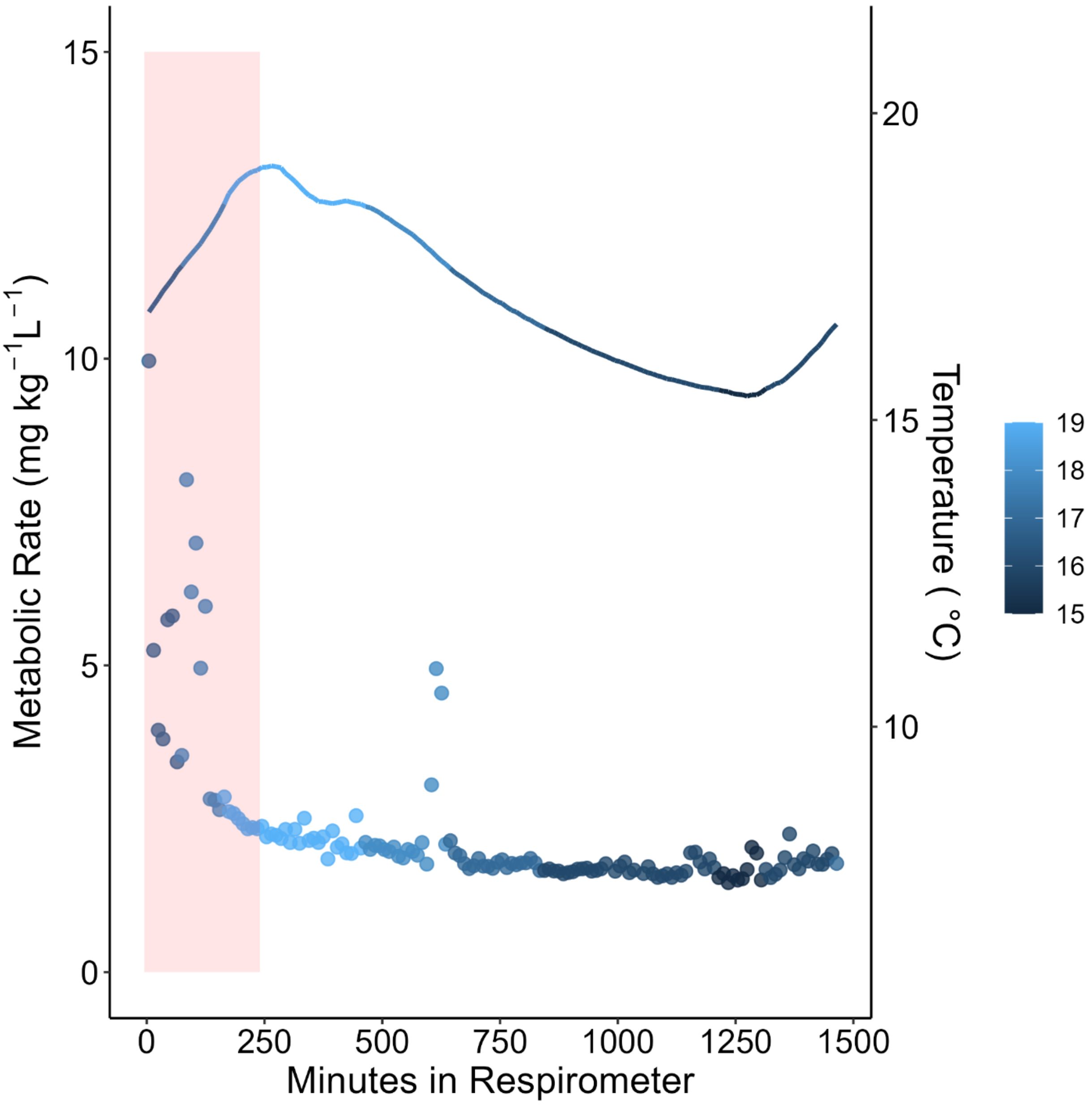

Figure S3. Resting metabolic rate (RMR) for each temperature within a common temperature treatment of 20-24°C.

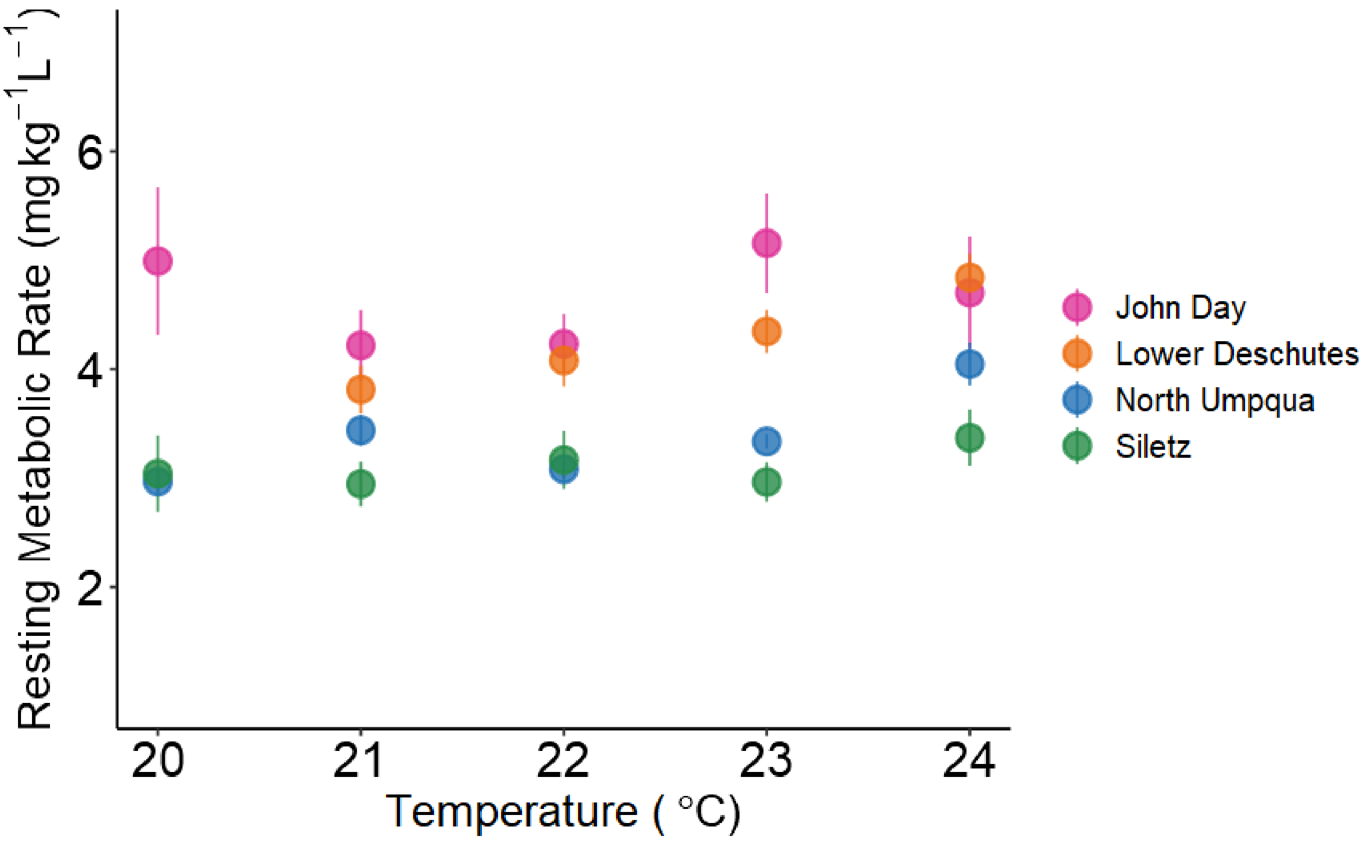

References

Killen SS, Christensen E., Cortese D, Závorka L, Norin T, Cotgrove L, Crespel A, Munson A, Nati JJ, Papatheodoulou M, McKenzie DJ (2021). Guidelines for reporting methods to estimate metabolic rates by aquatic intermittent-flow respirometry. *Journal of Experimental Biology*, 224(18).
